# Body aches, tender bones and rapid loss of weight: a case report

**DOI:** 10.1186/1757-1626-2-37

**Published:** 2009-01-10

**Authors:** Hadda Vijay, Vikram Kishore Navil, Jain Vaibhav, Anita Chopra, Ashish Goel, Rita Sood

**Affiliations:** 1 Department of Medicine, All India Institute of Medical Sciences, New Delhi 110029, India; 2 Department of Radiodiagnosis, All India Institute of Medical Sciences, New Delhi 110029, India; 3 Department of Haematopathology, All India Institute of Medical Sciences, New Delhi, India

## Abstract

**Introduction:**

Bone metastases presenting with pain and body-ache may be the first presentation of carcinoma in about a fourth of patients with cancer. Radiologically majority of the metastases are osteolytic and multiple. Sometimes these may be confused with infective or inflammatory conditions, particularly in young individuals, and degenerative conditions of the spine and hip in elderly, which may delay the diagnosis and treatment leading to poor outcomes.

**Case presentation:**

A 30 year old non-smoking male teetotaller presented with intermittent, high-grade nocturnal fever with night sweats of one year. He also had low back ache over his right hip. We found him febrile, pale and his long bones, ribs and pelvis were tender. He had a 3 × 4 cm tender and hard swelling over the upper part of his sternum. Another firm, non-tender swelling about 4 × 5 cm was seen in the right iliac region. Radiographs of the skull, spine and pelvis revealed multiple variable sized lytic lesions. A metastatic malignancy or disseminated tuberculosis was considered. His anti-tubercular therapy was intensified Fine needle aspiration from sternal lesion showed inflammatory cells. A bone marrow biopsy showed infiltration by tumor cells suggestive of metastatic adenocarcinoma. Patient's condition continued to deteriorate and he died within a fortnight of his hospitalization.

**Conclusion:**

Although masquerading as tuberculosis lytic lesions might be an evidence of malignant metastatic. Although, treatment is ineffective in this stage palliative efforts to improve quality of life should be made.

## Introduction

Multiple lytic lesions are a common radiological finding. Differential diagnoses are diverse and include infective, inflammatory and primary and metastatic malignancies. The skeletal system is the third most common site for distant metastases, following lung and liver. Bone metastases may be the first presentation of carcinoma in about 25 per cent of patients.[1] Pain is the common clinical presentation, which ranges from a dull ache to a deep, intense pain that is exacerbated by weight-bearing. Occasionally, the pain is worse at night and is not relieved by rest. Radiologically majority of the metastases are osteolytic and multiple. Sometimes these may be confused with infective or inflammatory conditions, particularly in young individuals, and degenerative conditions of the spine and hip in elderly, which may delay the diagnosis and treatment leading to poor outcomes. Here we present the case of a young man with multiple lytic bone lesions.

## Case presentation

A 30 year old non-smoking male patient presented with intermittent, high-grade nocturnal fever with night sweats of one year. He also had low back ache over his right hip. Prior to hospitalization, he had received four-drug anti-tubercular therapy (ATT) based on right sacro-ileitis seen on magnetic resonance imaging (MRI). No change in symptoms was observed during one year of therapy and he lost 10 kg weight. He also developed a tender swelling over sternum about a month before presenting to us. We found him febrile, pale and his long bones, ribs and pelvis were tender. He had a 3 × 4 cm tender and hard swelling over the upper part of his sternum. Another firm, non-tender swelling about 4 × 5 cm was seen in the right iliac region. He had mild anaemia with normal liver/renal functions, serum calcium and alkaline phosphatase.

**Figure 1:**
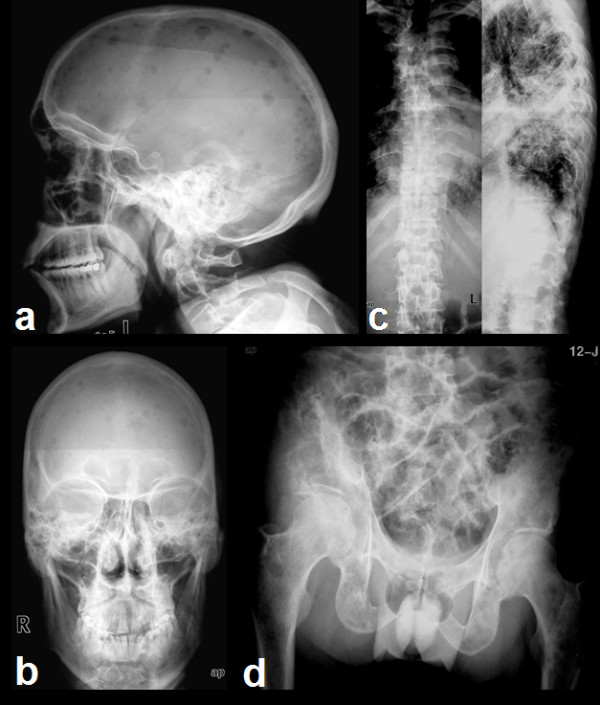
**Radiographs of the skull (a, b), spine (c), and pelvis (d) showing multiple variably sized lytic lesions affecting all visualized bones**.

**Figure 2:**
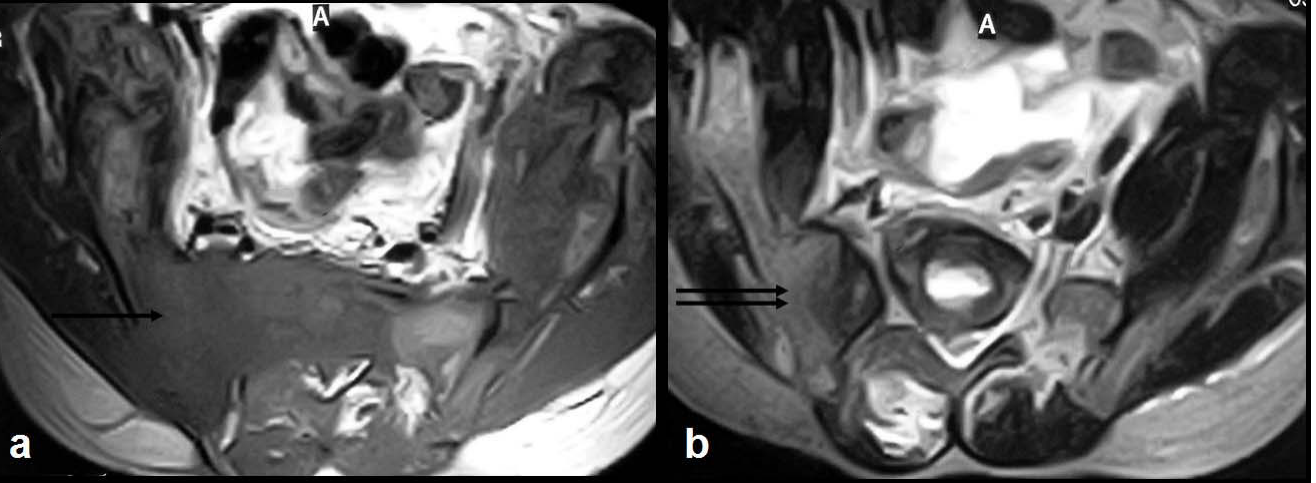
**Magnetic resonance imaging (MRI) T1W (a) image shows a hypointense mass lesion involving the right iliac bone extending into the sacrum (arrow)**. T2W MRI image (b) shows that the lesion in right iliac bone is hyperintense (double arrows).

Radiographs of the skull (Fig 1a and 1b), spine (Fig 1c) and pelvis (Fig 1d) revealed multiple variable sized lytic lesions affecting all visualized bones. T1 weighted MRI images of pelvis (Fig 2a) revealed a hypointense mass lesion involving the right iliac bone extending into the sacrum (arrow). T2 weighted MRI images (Fig 2b) revealed that the lesion in right iliac bone was hyperintense (double arrows).

**Figure 3:**
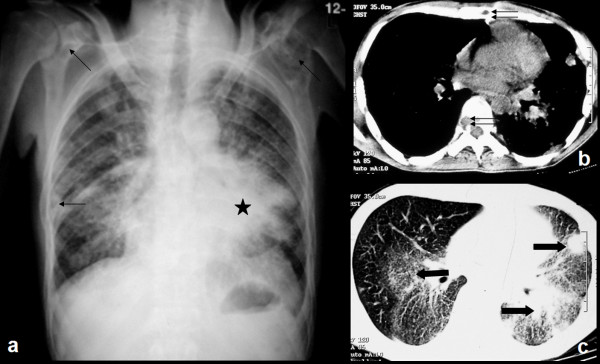
**Chest radiograph showing an ill-defined mass in the left mid and lower zones (star), alongwith multiple patches of consolidation in the right lung**. Multiple lytic bony lesions can also be seen (arrows). Computed tomography (CT) images (b, c) show the lung masses in detail (solid arrows). Also note the lytic lesions in the sternum and the vertebra (double arrows).

Plain chest radiograph (Fig 3a) of the patient revealed an ill-defined mass in the left mid and lower zones, along with multiple patches of consolidation in the right lung. Multiple lytic bony lesions were also seen (arrows). CT scan of chest (Fig 3b and 3c) showed the lung masses (solid arrows) and lytic lesions were observed in the sternum and the vertebra (double arrows).

**Figure 4:**
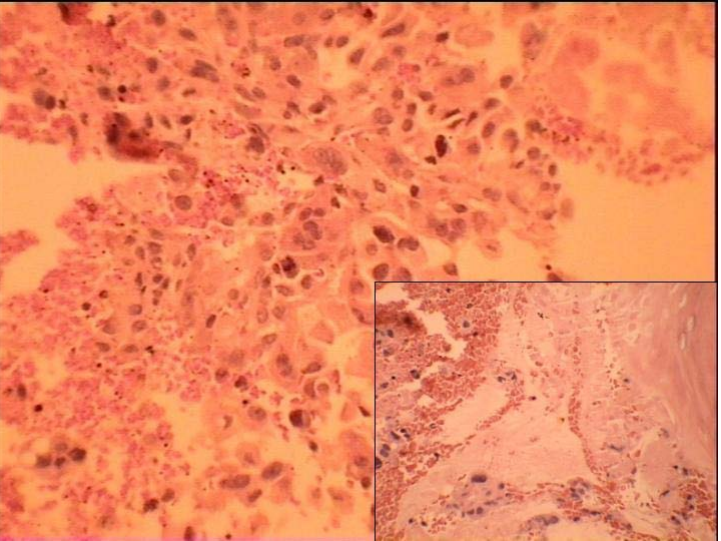
**Section examined from bone marrow biopsy showing a metastatic adenocarcinoma**.

A metastatic malignancy or disseminated tuberculosis was considered. His anti-tubercular therapy was intensified by addition of a quinolone and aminoglycoside to the existing regimen. Pain was controlled with NSAIDs. Fine needle aspiration performed from sternal lesion showed inflammatory cells. A bone marrow biopsy was taken from iliac crest and this showed infiltration of marrow spaces by tumor cells as shown in figure 4. The tumor was present in acinar architecture. The cells were polygonal in shape. The nuclei were hyperchromatic and showed moderate degree of nuclear pleomorphism. They had moderate amount of cytoplasm. The section also showed areas of procedural haemorrhage. These features are suggestive of metastatic adenocarcinoma. Patient's condition continued to deteriorate and he died within a fortnight of his hospitalization.

## Discussion

Bone is the favoured site for metastasis. Most metastases are osteolytic and multiple. Although several tumors like lung (10%), renal cell (10%) thyroid (5%) and adenocarcinoma (5%) are associated with osteolytic lesions, breast is the most common (50%).[2] The radiographic appearance of osteoarticular tuberculosis can mimic metastatic tumors or some primary osseous lesions, such as eosinophilic granuloma, especially if multiple destructive lesions are present.[3] The classical presentation of renal-cell carcinoma includes the triad of flank pain, hematuria, and a palpable abdominal mass in an adult male.[4] Lung carcinoma is seen usually in male smokers, although small cell carcinoma can be seen in non-smokers and also females. Tissue biopsy from lesion can differentiate between benign and malignant as well as the histopathological nature. In metastatic adenocarcinoma, as in this patient, identification of primary neoplasm proves difficult.[5] In our case, lung was the probable primary focus although this cannot be said with confirmation.

The treatment of metastatic bone disease consists of either systemic or local therapy depending upon patients' performance status. Systemic treatment can be chemotherapy, hormonal therapy, administration of radionuclides, or bisphosphonate therapy. The type of chemotherapy varies depending on the type of carcinoma. Bone lesions that progress during chemotherapy should be treated either with local irradiation or both operatively and with irradiation. The duration of survival after the diagnosis of metastatic bone disease often depends on the histological characteristics of the primary carcinoma. Patients who have metastatic bone disease secondary to breast carcinoma have a better prognosis for survival (34 months) than do those who have metastatic bone disease secondary to carcinoma of the prostate (24 months), cervix (18 months), colon and rectum (13 months), or lung (<12 months) or those who have it secondary to melanoma (about 3 months).[6,7]

Tuberculosis (TB) is endemic in this part of the world, involving 1.5% of our population.[8] Skeletal tuberculosis can present with articular/epiphyseal, articular/metaphyseal, metaphyseal without joint or flat bone involvement. It also can present as soft tissue swelling. The morphologic appearance can be similar to that of a lytic tumour or a destructive joint lesion. Soft-tissue TB presents as an abscess. On the basis of radiologic appearance, it can be difficult to differentiate peripheral osteoarticular and soft-tissue TB from other degenerative, inflammatory, or neoplastic disorders.[9] To prevent a delay in diagnosis, bone metastases should be considered in the differential diagnosis of multiple destructive skeletal lesions, even in young patients. If patient is not showing any improvement after about 6–8 weeks of ATT then alternate diagnosis should be strongly considered. In our patient ATT was continued for about 12 months despite no response and because of this crucial time was lost before a diagnosis of malignancy could be made. If diagnosed earlier, patient may have benefited from appropriate chemotherapy and/or radiotherapy.

## Conclusion

In conclusion, our case is unique because he taught us several lessons not only in the management of bone pains and body ache, an often neglected complain, but also in the humane care of a dying young patient. Although masquerading as tuberculosis lytic lesions might be metastatic lesions from a malignant source. Treatment is usually ineffective in this stage of disease. Palliative efforts to improve quality of life may go a long way in comforting the patient.

## Abbreviations

TB: Tuberculosis; CT: Computed tomography; MRI: Magnetic resonance imaging; ATT: Anti-tubercular therapy

## Consent

The patient expired during the course of his treatment during hospitalization. Further attempts to obtain consent from the patient's immediate family members and relatives have proved futile because they are not traceable.

## Competing interests

The authors declare that they have no competing interests.

## Authors' contributions

VH wrote the first draft of the manuscript. NKV provided intellectual inputs and was responsible for immediate patient care during hospitalization. AG provided continuous inputs and changes for modification to final manuscript and layout. RS was responsible for over-all patient care and provided final inputs in the manuscript. VJ analyzed and interpreted the patient data regarding the radiological picture. All authors read and approved the final manuscript.
